# Supplementary material for: Bacterial Colony Phenotyping with Hyperspectral Elastic Light Scattering Patterns
Source: Sensors (Basel). 2023 Mar 27;23(7):3485. doi: 10.3390/s23073485 (PMC10098818; doi:10.3390/s23073485)
Supplement: Supplementary file 1 [file sensors-23-03485-s001.zip › sensors-2239522-supplementary.pdf]

Bacterial colony phenotyping with hyperspectral elastic light scattering patterns

Iyll-Joon Doh[1], Diana Vanessa Sarria Zuniga[2], Sungho Shin[3], Robert E. Pruitt[2], Bartek Rajwa[4], J. Paul Robinson[3,5], and Euiwon Bae[1*]

[1]Applied Optics Laboratory, School of Mechanical Engineering, Purdue University, West Lafayette, Indiana, 47907, USA;
[2]Botany and Plant Pathology, Purdue University, West Lafayette, Indiana, 47907, USA;
[3]Basic Medical Sciences, College of Veterinary Medicine, Purdue University, West Lafayette, Indiana, 47907, USA;
[4]Bindley Bioscience Center, Purdue University, West Lafayette, Indiana, 47907, USA;
[5]Weldon School of Biomedical Engineering, Purdue University, West Lafayette, Indiana, 47907, USA

[*] Corresponding author

E-mail: ebae@purdue.edu (EB)

SUPPLEMENTARY SECTION

Table S1. The power of the diffracted beam with respect to the wavelength.

Wavelength (nm)	Drive Frequency (MHz)	Power (μW)
715.43	100	225.7
685.89	105	249.2
659.38	110	260.3
635.45	115	225.7
613.76	120	161.9
593.99	125	120.7
575.91	130	106.9
559.3	135	94.3
544	140	80.5
529.85	145	70.3
516.74	150	60.1
504.54	155	59.6
493.17	160	68.5
482.55	165	57.9
472.6	170	30.98
463.26	175	21.5
454.48	180	22.34

The overall power of the beam at VIS, reflected from the cold mirror, was measured at 65.6 mW.

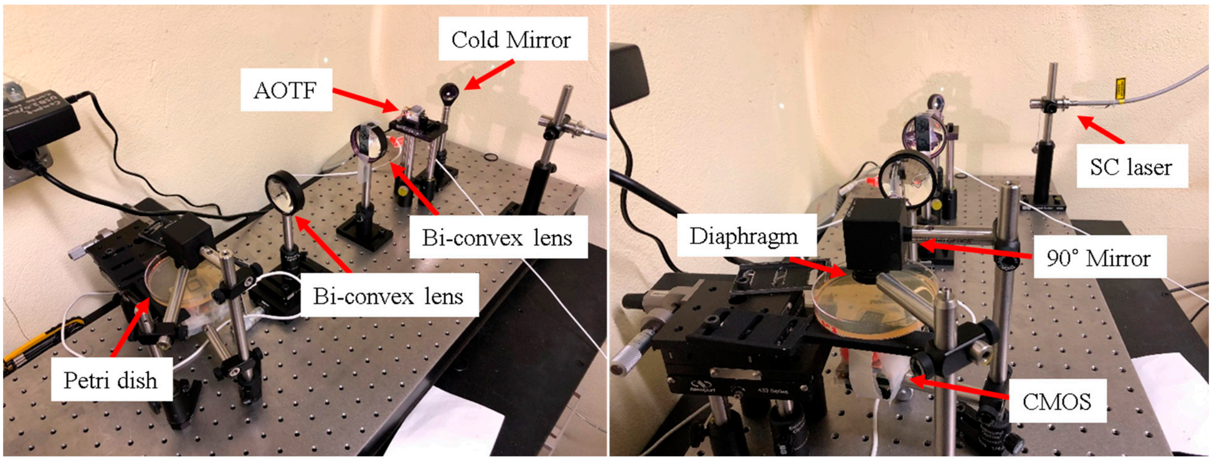

Figure S1. The experiment setup for the hyperspectral elastic light scatter phenotyping instrument (HESPI).

The picture of HESPI with the optical component are presented. As shown in the pictures, the bi-convex lenses were placed after the AOTF, and these components lay on the same axis. Owing to the position of the lenses, the size of the overall setup became about 25 inches long.

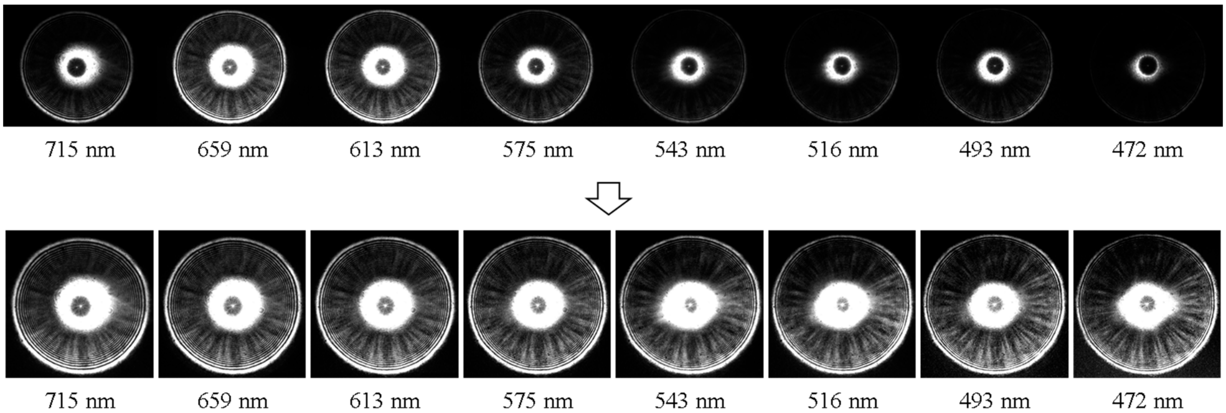

Figure S2. The contrast adjustment of the light scattering pattern for the intensity compensation.

For the image-processing, a contrast stretching function in MATLAB, "imadjust()" was utilized. The intensity values of each pattern image were adjusted to fill the entire dynamic range by stretching them based on the pattern image with the highest intensity. The dynamic range was set between 0 and the maximum intensity, adjusted by the ratio between the input image and the image with the highest intensity. Next, the lower and upper values of the input image were made to span the full dynamic range. All other intermediate values were reassigned to new intensity values according to the following formula.

$$g(x,y)=\frac{f(x,y)-f_{\min}}{f_{\max}-f_{\min}}\times 2^{bit}$$

Where $g(x,y)$ is the adjusted pixel value; $f(x,y)$ is input pixel value; $f_{\max}$ and $f_{\min}$ are upper and lower intensity values. The image was 8-bit gray scale image.

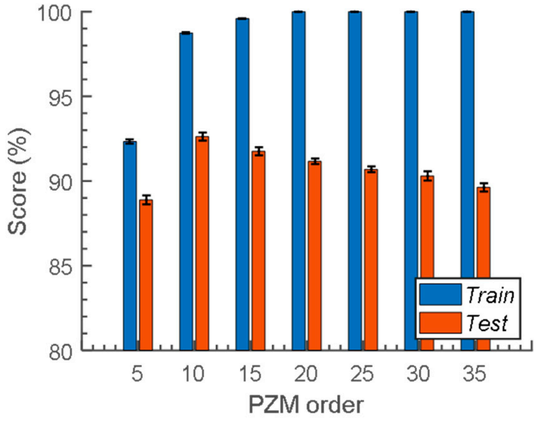

PZ order	Train Score (%)	Test Score (%)
5	92.3 (0.09)	88.9 (0.26)
10	98.7 (0.04)	92.6 (0.26)
15	99.6 (0.01)	91.8 (0.22)
20	100 (0)	91.2 (0.21)
25	100 (0)	90.7 (0.25)
30	100 (0)	90.3 (0.39)
35	100 (0)	89.6 (0.26)

Figure S3. The classification scores of the SVM-classifier with respect to the order of PZM (n=10).

The classification scores were the means of the true positive rates, and the overall average across the wavelengths was calculated. A linear SVM was utilized for the classifier since it has been traditionally used for the analysis of the ELS pattern. The order ranged from 5 to 35 with an increment of 5.

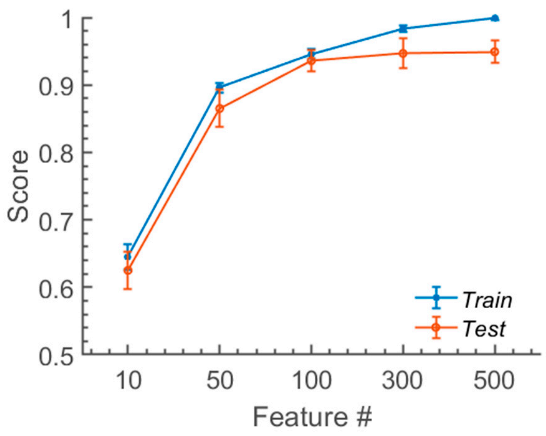

Figure S4. The classification score in relation to the number of features selected from the univariate feature selection.

Five different numbers of features were tested to determine the optimal number of features filtered from the univariate feature selection, specifically 10, 50, 100, 300, and 500 features. As shown on the graph, the training score increases as the number of features increases, while the test score reaches its peak when the number of features reaches 300. The error bar represents the standard deviation of 5 samples.

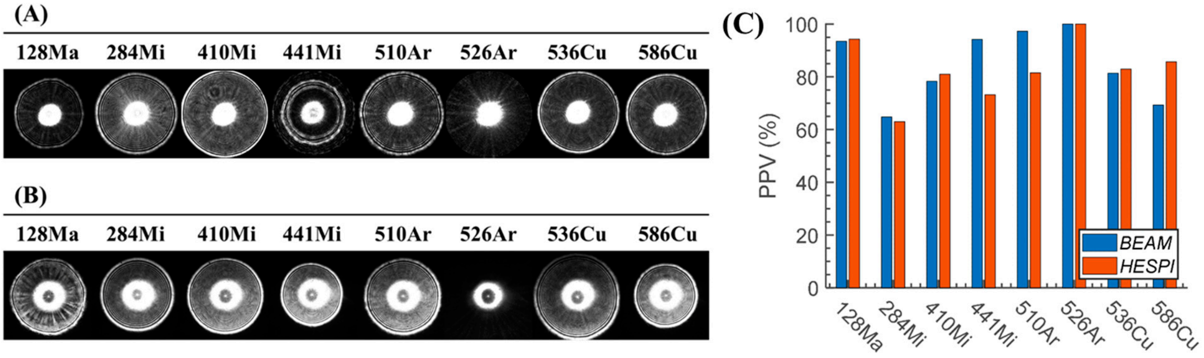

Figure S5. The classification of the sample bacteria using single wavelength method.

The representative light-scatter patterns of the bacteria collected from lettuce were measured using (A) a commercial ELS device (BEAM) and (B) HESPI with only a 635nm laser. Note that the incubation time was different. Panel (C) represents the classification result of 8 lettuce bacteria using only their 635-nm scattering pattern is presented. The 635-nm scattering patterns were measured using BEAM and HESPI. The result is given in positive predictive values (PPV) for each.

Table S2. The classification result is given in PPV for each wavelength. Green represents good, while red represents poor efficiency.

Wavelength (nm)	128Ma	284Mi	410Mi	441Mi	510Ar	526Ar	536Cu	586Cu
709	0.9118	0.8447	0.7890	0.9091	0.9551	0.9804	0.9787	0.9890
703	0.9159	0.8269	0.8447	0.8496	0.9892	1.0000	1.0000	0.9667
697	0.9065	0.8842	0.8396	0.7750	0.9892	0.9901	0.9889	0.9091
692	0.9327	0.8835	0.8713	0.8174	0.9892	1.0000	0.9468	0.9333
686	0.9151	0.8627	0.8889	0.8136	0.9894	1.0000	0.9674	0.9438
681	0.9510	0.8713	0.8364	0.8250	1.0000	1.0000	0.9778	0.9882
675	0.9208	0.8333	0.8529	0.8291	0.9891	1.0000	0.9783	0.9773
670	0.9118	0.8235	0.7909	0.8435	1.0000	1.0000	0.9890	0.9886
665	0.9208	0.8019	0.8073	0.8348	1.0000	1.0000	0.9674	0.9884
660	0.9485	0.8000	0.8269	0.8496	0.9785	1.0000	0.9574	0.9775
655	0.9184	0.8700	0.8447	0.8362	0.9684	1.0000	0.9485	0.9560
650	0.9175	0.8095	0.8252	0.8649	0.9894	1.0000	0.9381	0.9570
645	0.9474	0.8037	0.7636	0.8584	0.9667	1.0000	0.9684	0.9667
640	0.9200	0.8039	0.8095	0.8649	0.9789	1.0000	0.9574	0.9785
636	0.9010	0.8165	0.8713	0.8571	0.9785	1.0000	0.9785	0.9780
631	0.9388	0.8273	0.8317	0.8559	0.9684	1.0000	0.9677	0.9565
627	0.9010	0.8200	0.7944	0.8435	0.9787	1.0000	0.9570	0.9667
622	0.8922	0.8113	0.8182	0.8496	0.9785	1.0000	0.9462	0.9468
618	0.9100	0.7982	0.8280	0.8348	0.9588	1.0000	0.9583	0.9667
614	0.9388	0.8173	0.8542	0.8571	0.9697	1.0000	0.9684	0.9167
610	0.9184	0.7963	0.8421	0.8818	0.9700	1.0000	0.9579	0.9468
606	0.9278	0.8302	0.8660	0.8696	0.9592	1.0000	0.9485	0.9778
602	0.9223	0.8673	0.8542	0.8534	0.9314	1.0000	0.9681	0.9780
598	0.9208	0.8713	0.9175	0.8750	0.9505	1.0000	0.9574	0.9468
594	0.8942	0.8431	0.8725	0.8829	0.9894	1.0000	0.9579	0.9565
590	0.9314	0.8700	0.8738	0.8850	0.9592	1.0000	0.9674	0.9674
586	0.8932	0.8696	0.8611	0.8305	0.9691	1.0000	0.9570	0.9663
583	0.9223	0.8529	0.8846	0.8621	0.9684	1.0000	0.9670	0.9663
579	0.9684	0.8854	0.8598	0.8333	0.9293	1.0000	0.9681	0.9663
576	0.9135	0.8600	0.8824	0.8727	0.9479	1.0000	0.9362	0.9362
572	0.9688	0.8889	0.9100	0.8376	0.9307	1.0000	0.9479	0.9560
569	0.9691	0.8958	0.8952	0.8522	0.9400	1.0000	0.9574	0.9570
565	0.9300	0.8788	0.9126	0.8584	0.9216	1.0000	0.9674	0.9560
562	0.9787	0.9043	0.9057	0.8761	0.9091	1.0000	0.9388	0.9583
559	0.9792	0.8763	0.9314	0.8696	0.9327	1.0000	0.9787	0.9457
556	0.9500	0.8654	0.9505	0.9340	0.9216	1.0000	0.9674	0.9474
553	0.9688	0.8558	0.9238	0.9151	0.9505	1.0000	0.9579	0.9677
550	0.9794	0.8911	0.9327	0.9434	0.9412	1.0000	0.9787	0.9583
547	0.9897	0.8725	0.9505	0.9259	0.9900	1.0000	0.9490	0.9468
544	0.9898	0.8922	0.9802	0.8761	0.9800	1.0000	0.9792	0.9778
541	0.9897	0.8713	0.9706	0.8839	0.9706	1.0000	0.9792	0.9667
538	1.0000	0.8416	0.9519	0.8899	0.9900	1.0000	0.9691	0.9355
535	0.9898	0.8431	0.9796	0.9245	0.9899	1.0000	0.9400	0.8969
532	1.0000	0.8529	0.9608	0.9252	0.9612	1.0000	0.9688	0.9457
529	1.0000	0.8788	0.9612	0.9009	0.9804	1.0000	0.9691	0.9457
527	0.9898	0.8515	0.9612	0.9174	0.9340	1.0000	0.9780	0.9348
524	1.0000	0.8866	0.9340	0.9091	0.9804	1.0000	0.9789	0.9355
522	1.0000	0.8788	0.9596	0.9091	0.9615	1.0000	0.9490	0.9457
519	0.9798	0.9010	0.9802	0.9091	0.9789	1.0000	0.9200	0.9468
516	1.0000	0.8911	0.9706	0.9009	0.9596	1.0000	0.9286	0.9560
514	0.9898	0.8922	0.9596	0.9259	0.9804	1.0000	0.9583	0.9474
511	0.9694	0.9091	0.9700	0.9346	0.9417	1.0000	0.9278	0.9271
509	0.9694	0.8545	0.9703	0.9434	0.9510	1.0000	0.9677	0.9556
507	0.9588	0.9208	0.9703	0.9009	0.9314	1.0000	0.9468	0.9362
504	0.9505	0.9184	0.9615	0.9174	0.9583	0.9804	0.9574	0.9271
502	0.9394	0.9072	0.9608	0.9174	0.9208	1.0000	0.9574	0.9388
500	0.9175	0.8800	0.9510	0.9000	0.9118	1.0000	0.9574	0.9579
497	0.9200	0.8687	0.9608	0.9151	0.9192	1.0000	0.9474	0.9091
495	0.9126	0.8627	0.9510	0.8972	0.9468	1.0000	0.9474	0.9175
493	0.8932	0.8447	0.9406	0.8818	0.9263	1.0000	0.9474	0.9355
491	0.9057	0.8173	0.9412	0.8972	0.9659	1.0000	0.9381	0.9167
489	0.9151	0.9158	0.9423	0.8879	0.9468	1.0000	0.9375	0.8878
487	0.9065	0.8900	0.9798	0.8879	0.9394	1.0000	0.9674	0.8958
485	0.9020	0.8350	0.9500	0.8704	0.9362	1.0000	0.9479	0.9072
483	0.9208	0.8476	0.9381	0.9238	0.9200	1.0000	0.9677	0.9192
481	0.9223	0.8713	0.9505	0.9143	0.9184	1.0000	0.9574	0.9082
479	0.8932	0.7981	0.9118	0.9009	0.9457	1.0000	0.9684	0.9462
477	0.9118	0.8515	0.9126	0.9074	0.9457	1.0000	0.9381	0.9485
475	0.8922	0.8462	0.9500	0.9174	0.9468	1.0000	0.9574	0.9588
473	0.9388	0.8476	0.9515	0.9174	0.9677	1.0000	0.9388	0.9574

Table S3. Classification performance of eight bacterial species utilizing the elastic net logistic regression classifier created with hyperspectral ELS data. (n=10).

	128Ma	284Mi	410Mi	441Mi	510Ar	526Ar	536Cu	586Cu
**Accuracy**	98.65 (0.76)	98.23 (1.57)	99.48 (0.62)	99.58 (0.45)	99.48 (0.62)	100.00 (0.00)	98.13 (0.97)	98.33 (0.63)
**Sensitivity**	93.33 (5.04)	95.83 (4.96)	97.50 (4.96)	100.00 (0.00)	97.50 (3.45)	100.00 (0.00)	92.50 (6.61)	90.83 (7.07)
**Specificity**	99.40 (0.87)	98.57 (1.53)	99.76 (0.44)	99.52 (0.51)	99.76 (0.44)	100.00 (0.00)	98.93 (0.79)	99.40 (0.71)
**PPV**	96.13 (5.60)	91.27 (8.79)	98.44 (2.89)	96.88 (3.34)	98.39 (2.99)	100.00 (0.00)	92.77 (5.10)	96.02 (4.57)
**NPV**	99.06 (0.71)	99.40 (0.70)	99.65 (0.70)	100.00 (0.00)	99.65 (0.49)	100.00 (0.00)	98.94 (0.93)	98.71 (0.99)

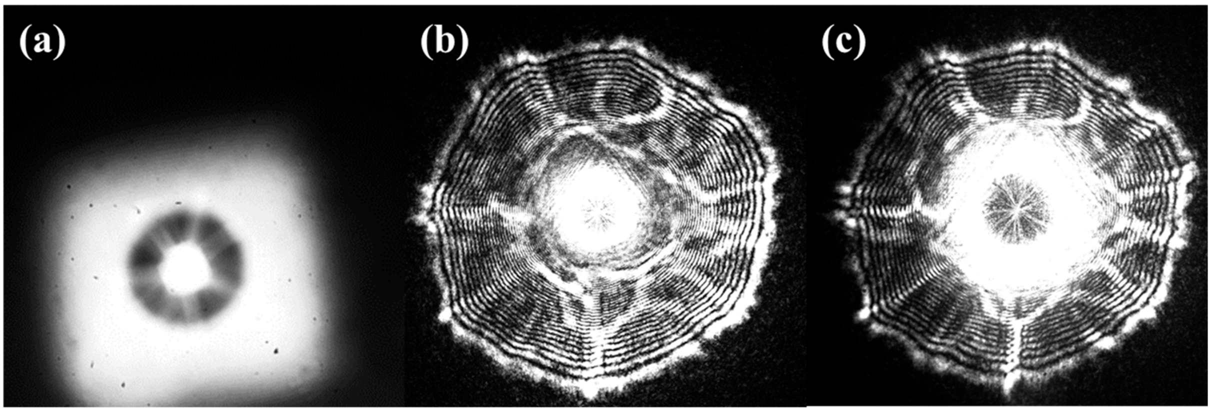

Figure S6. The light-scatter pattern generation with different coherence properties. Three light sources were utilized to create the light-scatter pattern from the E. coli K12 colony: (a) LED flashlight as an incoherent light source, (b) 635 nm laser diode as a coherent light source, and (c) HESPI as the unknown.

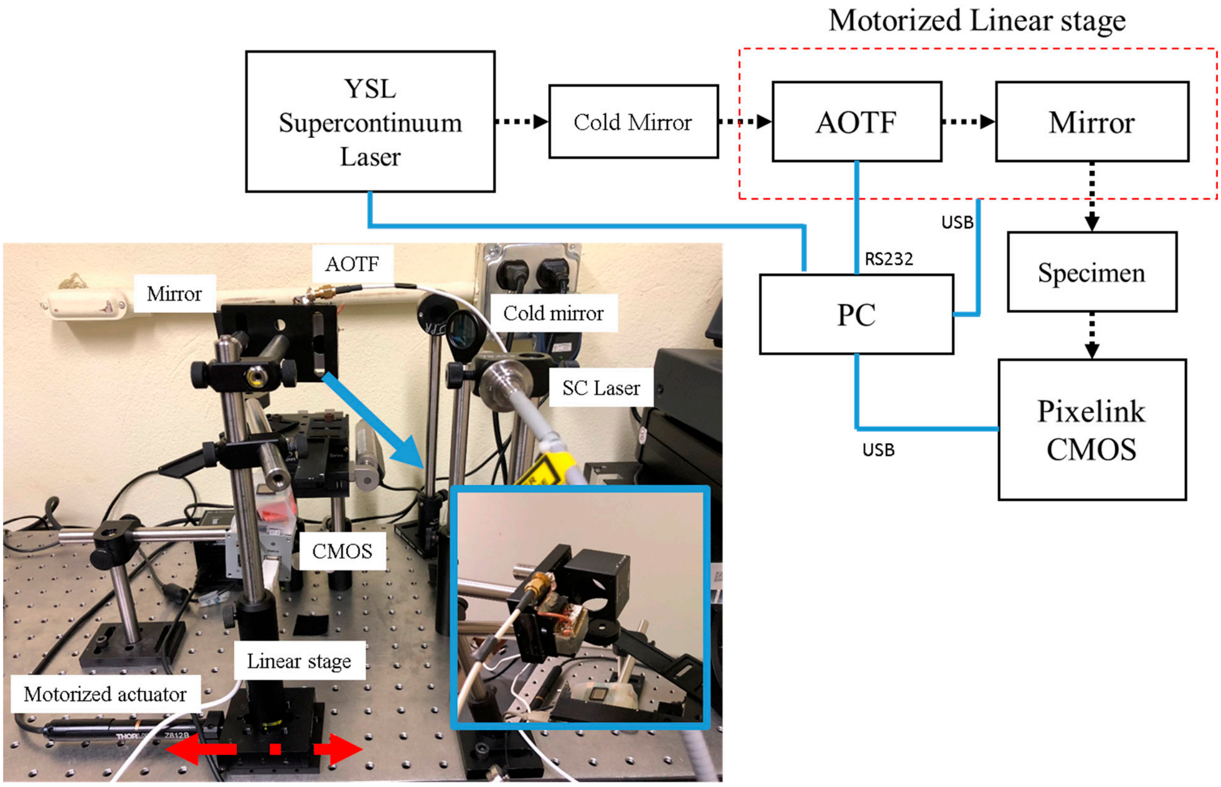

Figure S7. The schematic diagram and picture of HESPI with a mechanical solution to compensate for the beam spot movement at the imaging plane.

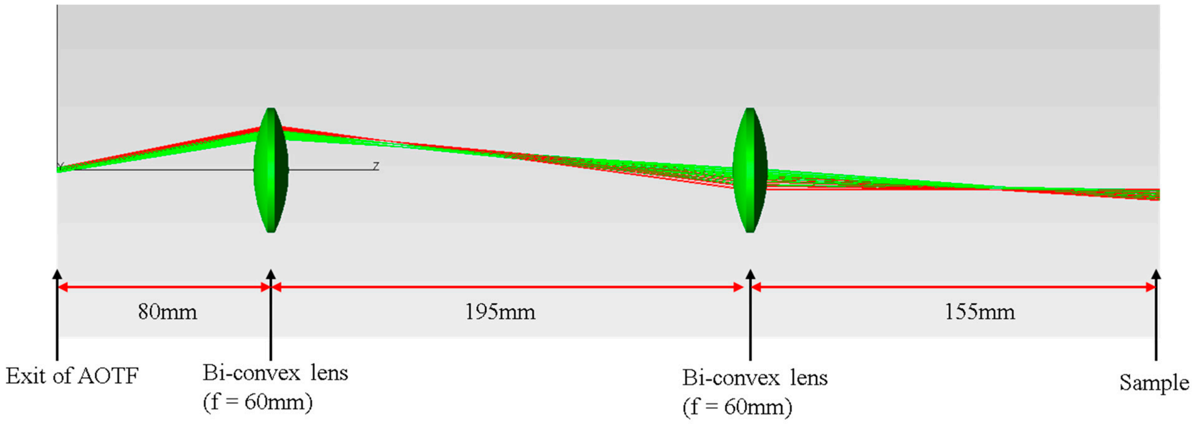

Figure S8. A simple ray-tracing simulation of multiple lenses implemented in HESPI to minimize the beam spot movement at the imaging plane.
